# Novelties in the ISO 15189:2023 standard

**DOI:** 10.1515/almed-2023-0139

**Published:** 2023-11-13

**Authors:** Isabel de la Villa Porras

**Affiliations:** Head of ENAC Healthcare Section, Madrid, Spain

**Keywords:** ISO 15189 standard, quality, clinical laboratories

In December 2022, the International Standard Organization published the update of the ISO 15189 standard “*Clinical Laboratories: Requirements for Quality and Competence*” [1]. After a minimal revision of the Standard in 2007, the first update was published in 2012 for clarification purposes [2]. The 2022 update modifies the structure of the Standard and establishes requirements for point-of-care testing (POCT). Twenty years after the first version of the ISO 15189 standard was published, the Standard has became the global gold-standard method for guaranteeing the correct performance of clinical laboratories via accreditation.

Professionals of all specialties from all over the world have participated in the development and revision of the ISO 15189 Standard through the International Standardization Structure, ISO. This structure is made up with of members from the national standardization bodies, in Spain Asociación Española de Normalización, UNE, www.une.org through the UNE’s National Technical Committee, CTN 129 for the ISO 15189. This CTN integrates different scientific societies, including the Spanish Society of Laboratory Medicine (SEQC-ML). The ISO structure is based on consensus from experts representing the interested parties which adds value and guarantees international validity and recognition.

The current update only contains minor changes to ISO 15189 requirements, which demonstrates the high level of development and consensus about its contents. In addition, this update guarantees the validity of most systems implemented in clinical laboratories under the former version. As a result, the new version does not entail significant modifications in ISO-accredited laboratories.

The changes have been mainly made to the structure of the Standard, in line with the ISO/IEC 17025:2017 Standard [3]. This new version also incorporates point-of-care testing (POCT) requirements, which are now an integral part of the Standard and supersede ISO 22870:2016 [4].

The new version of the Standard puts more emphasis on aspects already considered in the 2012 version, including patient safety, risk based approach, and the promotion of continuous improvement.

A remarkable aspect of this version is the less prescriptive formulation of the text, thereby providing higher flexibility in complying and proving conformity with Standard requirements. This formulation enables better adaptation for any type of test or clinical laboratory discipline.

As a result of the increasing number of ISO standards recently published in the healthcare sector, this Standard makes reference to several ISO clinical laboratory standards (i.e. ISO 22367 – *Application of risk management in the clinical laboratory*, ISO 20658:2023 – *Requirements for the collection and transport of samples intended for clinical laboratory analysis*).

This new version will require accredited and applicant clinical laboratories and to adapt their systems to ISO requirements. For that purpose, ENAC established a transition period (in compliance with the requirements of the *International Laboratory Accreditation Cooperation*, ILAC) that will end in December 6, 2025, when all effective clinical laboratory accreditations will be required to make reference to the new version of the Standard.

Before that date, all laboratories will have to prove compliance with ISO 15189:2022 requirements. Considering that ISO 15189:2022 incorporates ISO 22870:2016 requirements, once POCT-accredited laboratories have demonstrated compliance with the new requirements, their scope of accreditation will be updated, and the reference to ISO 22870:2016 will be removed.

## ISO 15189 accreditation of clinical laboratories

Accreditation is an internationally accepted tool for the evaluation of compliance with the Standard. This independent, rigorous mechanism generates confidence and credibility on the correct performance of specific types of activities.

In Spain, the evaluation process is performed by ENAC (national accreditation entity, www.enac.es). To ensure that the evaluation process was completely effective, ENAC joined the global accreditation infrastructure (in Europe, EA, and at international level, ILAC) 30 years ago. These structures establish the international criteria and harmonization mechanisms to be adopted by all accreditation entities.

Additionally, ENAC is signatory of the mutual recognition agreements through a “peer evaluation” system, by which all parties recognize and accept each other’s accredited reports to stay true to their motto: “*Accredited once, accepted everywhere*”.

To ensure harmonized evaluation of conformity with the ISO 15189 standard, ENAC participates in the European and global healthcare working groups, together with representatives form other accreditation entities, European scientific societies and the *in vitro* diagnostics industry.

ENAC collaborates with scientific societies of each discipline for via Collaboration Agreements, which were signed by SEQC in 2005.
